# Dual ECM Biomimetic Scaffolds for Dental Pulp Regenerative Applications

**DOI:** 10.3389/fphys.2018.00495

**Published:** 2018-05-25

**Authors:** Chun-Chieh Huang, Raghuvaran Narayanan, Noah Warshawsky, Sriram Ravindran

**Affiliations:** ^1^Department of Oral Biology, University of Illinois at Chicago, Chicago, IL, United States; ^2^Department of Endodontics, University of Illinois at Chicago, Chicago, IL, United States

**Keywords:** dental pulp regeneration, biomimetic scaffolds, extracellular matrix, dental pulp stem cells, pro-angiogenic matrix

## Abstract

Dental pulp is a highly vascularized and innervated tissue that provides sensitivity and vitality to the tooth. Chronic caries results in an infected pulp tissue prone to necrosis. Existing clinical treatments replace the living pulp tissue with a non-responsive resin filling resulting in loss of tooth vitality. Tissue engineering approaches to dental pulp tissue regeneration have been investigated to preserve tooth vitality and function. However, a critical criterion is the choice of growth factors that may promote mesenchymal stem cell differentiation and more importantly, vascularization. But, the problems associated with growth factor dosage, delivery, safety, immunological and ectopic complications affect their translatory potential severely. The purpose of this study is to develop, characterize and evaluate a biomimetic native extracellular matrix (ECM) derived dual ECM scaffold that consists of a pulp-specific ECM to promote MSC attachment, proliferation and differentiation and an endothelial ECM to promote migration of host endothelial cells and eventual vascularization *in vivo*. Our results show that the dual ECM scaffolds possess similar properties as a pulp-ECM scaffold to promote MSC attachment and odontogenic differentiation *in vitro*. Additionally, when implanted subcutaneously in a tooth root slice model *in vivo*, the dual ECM scaffolds promoted robust odontogenic differentiation of both dental pulp and bone marrow derived MSCs and also extensive vascularization when compared to respective controls. These scaffolds are mass producible for clinical use and hence have the potential to replace root canal therapy as a treatment for chronic dental caries.

## Introduction

Dental caries is a widely prevalent infectious disease second only to the common cold (Islam et al., 2007). About 90% of the world’s population has experienced dental caries (WHO report). Caries is characterized by loss of mineralized tissues of the teeth (enamel and dentin) owing to acidity caused by bacterial growth. Progression of this condition exposes the soft dental pulp tissue leading to infection of the pulp causing pain, distress and loss to quality of life. In its advanced stage, dental caries results in inflammation and necrosis of the vital dental pulp tissue.

The dental pulp is a highly vascularized and innervated tissue that provides sensitivity, vitality, immunoprotective and regenerative ability to the tooth. In addition, the dental pulp also serves as a source for mesenchymal stem cells (MSCs) (Tirino et al., 2011, 2012). Maintenance of a healthy, vascularized and innervated dental pulp is necessary for a healthy tooth. Although poor oral hygiene is the primary cause for dental caries, cancer treatments such as radiation therapy and chemotherapy as well as medications that affect salivation and dry-mouth syndrome also can cause increase in oral bacteria leading to acute and chronic dental caries.

Advanced stages of dental caries that involve necrotic pulp tissue are often treated by root canal therapy. Several million root canal treatments are performed every year around the world and approximately 20 million in the United States alone. Although this process is effective, it results in the treated tooth loosing its vitality and sensitivity. This loss in sensitivity results in future infections going unchecked until it spreads to surrounding tissues creating an acute or chronic condition requiring significant intervention and therapy and a considerable loss to the quality of life. In adolescents, root canal therapy prevents root maturation that can lead to cervical root fractures (Cvek, 1992).

These ill effects can be avoided if the dental pulp tissue can be regenerated using tissue-engineering approaches. However, dental pulp tissue regeneration is challenging owing to the difficulties in getting engineered tissues vascularized through the narrow root canal cavity at the distal end of the root. Traditional approaches to dental pulp tissue engineering utilize growth factors (Nakashima, 1994; Nakashima and Reddi, 2003; Morito et al., 2009), and biomaterials as cues to achieve mesenchymal stem cell differentiation and vascularization (Gronthos et al., 2002; Cordeiro et al., 2008; Demarco et al., 2010; Huang et al., 2010; Karaoz et al., 2011; Rosa et al., 2013). The primary drawback with such approaches is the dosage, safety, delivery and side effects associated with growth factor usage. These approaches utilize a single morphogen system that dictates the use of the morphogen at physiologically irrelevant high levels that can lead to complications clinically [as observed in the case of BMP2 usage for bone regeneration (Hustedt and Blizzard, 2014)].

Alternatively, some groups have evaluated a cell free approach that focuses on the use of growth factors as cell-homing signals (Kim J.Y. et al., 2010; Kim K. et al., 2010; Yang et al., 2015). These elegant approaches using ectopic implantation models have shown vascularization and reparative dentin formation. More recently, the use of chemokines for host stem cell homing has also been evaluated for pulp regeneration (Pan et al., 2013; Takeuchi et al., 2015). However, these studies like the previous ones also deal with non-physiological levels of growth factors and possible side effects of those growth factors. On the other hand, they iterate the importance of the presence of signaling molecules in the pulp scaffolds to facilitate host cell migration and vascularization.

Published studies by ours and other groups shown that collagen scaffolds embedded with lineage-specific extracellular matrix (ECM) can induce odontogenic, osteogenic, and chondrogenic differentiation of naïve MSCs *in vitro* and *in vivo* depending on the nature of the ECM (Ravindran et al., 2012, 2015a,b; Ravindran and George, 2015; Paduano et al., 2017). With vascularization being necessary criteria for pulp regeneration and with several studies highlighting the importance of cell homing, we hypothesized that a dual ECM scaffold consisting of a dental pulp cell ECM in combination with an endothelial cell ECM might not only promote stem cell differentiation, but also promote enhanced endothelial cell home and vascularization. In the present manuscript, we have developed such a scaffold and characterized it in terms of composition and performance both *in vitro* and *in vivo*.

The goal of this study was to evaluate the potential of the dual ECM scaffolds side-by-side with pulp ECM scaffolds to compare their ability to promote vascularization as well as to evaluate if the dual ECM scaffolds possessed the properties of the pulp ECM scaffolds to promote MSC attachment, proliferation and differentiation.

## Materials and Methods

### Cell Culture

Two mesenchymal stem cell types were used in this study namely: Primary human dental pulp MSCs (DPSCs) and primary human bone marrow derived MSCs (HMSCs). DPSCs were a gift from Dr. Songtao Shi (University of Pennsylvania, School of Dental Medicine). HMSCs used in this study were purchased from ATCC. Both types of MSCs were cultured in MEM-alpha containing 20% fetal bovine serum (Gibco), 1% antibiotic-antimycotic solution (Gibco) and 1% L-Glutamine (Gibco). The cells were not passaged our used beyond passage 4. For experiments that required odontogenic differentiation of the cells, growth media was supplemented with 100 μg/ml ascorbic acid, 10 mM β-glycerophosphate and 10 mM dexamethasone.

Human umbilical vein endothelial cells were used to generate a pro-vascular ECM. These cells were cultured in F12K medium (Gibco) supplemented with 10% FBS (Gibco), heparin (Sigma) and endothelial growth supplement (Sigma) as per previously published protocols (Ravindran et al., 2004).

### Generation of ECM Scaffolds

A collagen-chitosan base hydrogel was used as the starting material for the generation of ECM embedded scaffolds. Briefly, DPSCs were embedded homogenously (1 × 10^6^ cells/ml of hydrogel) within collagen chitosan hydrogels (500 μl monomer suspension containing 500,000cells) and the pulp ECM scaffold was generated by decellularization after a 2-week culture of the DPSCs within the hydrogel under the influence of odontogenic differentiation media as per our previously published protocols (Ravindran et al., 2014a,b). Briefly, for decellularization, the scaffolds containing cells were treated with TBS containing triton X-100 to permeablize the cells followed by cell lysis in 25 mM ammonium hydroxide after which the cells were washed in HEPES buffered saline. This was followed up with DNAse treatment of the scaffolds to remove DNA bound to the scaffold from the lysed cells. The dual ECM scaffolds were generated with the pulp ECM scaffolds as the base material. For the generation of the dual ECM scaffolds, HUVECs were seeded within the decellularized pulp ECM scaffolds (500,000 cells/scaffold) and the constructs were cultured in HUVEC culture medium for 1 week. The scaffolds were decellularized using the same protocol described above followed by DNAse treatment as per our published protocols (Ravindran et al., 2014a,b). The scaffolds thus generated were washed in double deionized water, lyophilized and stored in sterile containers at room temperature until further use.

### Immunohistochemical Characterization of ECM Scaffolds

The presence of various ECM proteins within the scaffolds was evaluated by immunohistochemistry. Briefly, the control (pulp ECM) and the dual ECM scaffolds were fixed in neutral buffered 4% formalin, embedded in paraffin and sectioned into 5 μm thick sections. The sections were then fluorescently immunostained using the following antibodies: rabbit polyclonal anti fibronectin (1/500, Abcam), mouse monoclonal anti dentin matrix protein 1 (DMP1, 1/1000, kind gift from Dr. Anne George, UIC), rabbit polyclonal anti Dentin phosphophoryn (DPP, 1/100, kind gift from Dr. Anne George, UIC), rabbit polyclonal anti Dentin sialoprotein (DSP, 1/100, kind gift from Dr. Anne George), rabbit polyclonal anti transforming growth factor beta 1 (TGFβ1, 1/100, Abcam), mouse monoclonal anti bone morphogenetic protein 2 (BMP2, 1/100, Abcam), mouse monoclonal anti von Willebrand factor (vWF, 1/100, Abcam), mouse monoclonal anti vascular endothelial growth factor (VEGF, 1/100, Abcam), rabbit polyclonal anti basic fibroblast growth factor (bFGF, 1/100, Abcam) followed by TRITC conjugated anti rabbit and FITC conjugated anti mouse secondary antibodies as required.

### Proliferation Experiments

Proliferation of DPSCs and HMSCs on pulp ECM and dual ECM scaffolds were performed as per previously published protocols (Ravindran et al., 2014a). Briefly, 20,000 cells were seeded on to the respective scaffolds placed in 96 well assay plates and the increase in cell number was measured over a period of 10 days using the cell-titer kit (Promega). All experiments were performed in quadruplicates. Results are presented as mean ± SD. Student’s *t*-test was used to measure statistical significance.

### Real Time Quantitative PCR

The expression levels of odontogenic marker genes in DPSCs and HMSCs cultured within the pulp and dual ECM scaffolds were evaluated by qRT PCR as per previously published protocols (Ravindran et al., 2014a,b, 2015b). Briefly, 100,000 cells were cultured within control (no ECM), pulp or dual ECM scaffolds in quadruplicates and cultured for 7 days in the presence of standard growth media. RNA from the cells was isolated using standardized kits (Qiagen) followed by cDNA synthesis and qPCR for selected marker genes using gene specific primers published from our previous studies (Ravindran et al., 2014a,b, 2015b). All experiments were performed in triplicate and fold change was calculated using the DDCt method and Student’s *t*-test was performed to evaluate statistical significance between the respective group and the corresponding control group.

### Tooth Root Slice Subcutaneous Implantation Model

All animal surgeries were performed as per UIC Animal Care Committee (ACC) approved protocols (Protocol no: 16-001, Institutional assurance no: A3460-01). Tooth root slices were generated from discarded human teeth extracted for orthodontic purposes from the UIC clinics. The root slices were a kind gift from Dr. Satish Alapati of the UIC Endodontics department. Experiments were performed as per previously published methods (Huang et al., 2016). Briefly, 100,000 cells (DPSC or HMSCs) were seeded on to the respective control, pulp or dual ECM scaffolds. The root canal spaces of the root slices were then filled with the respective scaffolds and implanted subcutaneously bilaterally on to the back of immunocompromised mice for a period of 4 weeks. Plain collagen scaffolds containing DPSCs or HMSCs served as the control group and the pulp and dual ECM scaffolds served with DPSCs or HMSCs served as the experimental group. Each group contained four replicates. The samples were then extracted, fixed in neutral buffered 4% formalin, decalcified in EDTA solution, embedded in paraffin, sectioned into 5 μm thick sections and subjected to histology or immunohistochemistry (IHC).

## Results

### Characterization of the Dual ECM Scaffolds

**FIGURE 1:**
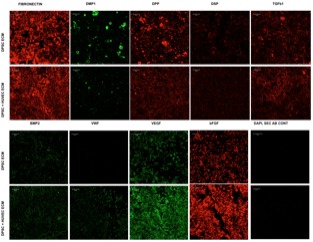
Immunohistochemical characterization of the dual ECM scaffolds. The figures are representative confocal micrographs of pulp ECM (DPSC ECM) and dual ECM (DPSC+HUVEC ECM) scaffolds immunostained for several markers for ECM proteins. The images are split into two groups and arranged vertically based on antibody labeling. Note the increased presence of pro-vascular proteins such as von Willebrand factor (VWF), VEGF and bFGF. Scale bar represents 20 μm in all images.

The purpose of generating the dual ECM scaffolds is to enhance the vascularization potential without adversely affecting MSC attachment, proliferation and differentiation potential. Therefore, our first step was targeted at verifying the presence of various ECM proteins that serve as potential indicators of scaffold function. The presence of ECM proteins necessary for odontogenic differentiation of MSCs as well as those that are important for vascularization was evaluated by immunohistochemistry in the pulp ECM and the dual ECM scaffolds. Results presented in **Figure 1** indicate that both types of scaffolds contained proteins required for MSC differentiation such as Fibronectin, dentin matrix protein 1 (DMP1), dentin phosphophoryn (DPP), dentin sialoprotein (DSP), and bone morphogenic protein 2 (BMP2). The dual ECM scaffolds, however, showed a reduced level of expression with respect to DMP1, DSP, and DPP. On the other hand, presence of proteins such as von Willebrand factor (vWF), vascular endothelial growth factor (VEGF) and basic fibroblast growth factor (bFGF) were present in increased levels in the dual ECM scaffolds indicating that these scaffolds possessed higher levels of proteins that promote vascularization and angiogenesis.

### Proliferation of DPSCs and HMSCs

**FIGURE 2:**
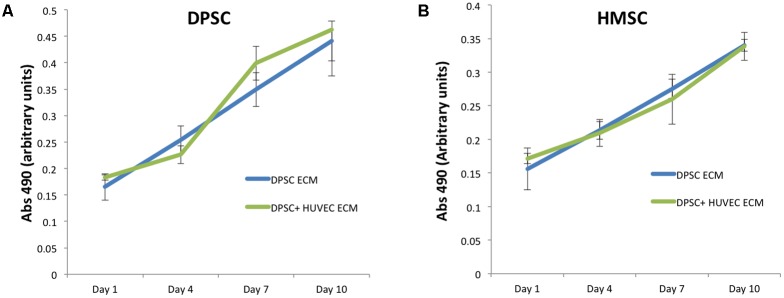
Proliferation of DPSC and HMSC in the pulp and dual ECM scaffolds. **(A)** Graph represents increase in absorbance at 490 from the MTS assay indicating an increase of DPSC cell number over time in the pulp (DPSC ECM, blue line) and dual (DPSC+HUVEC ECM, green line) scaffolds. **(B)** Graph represents similar data for HMSCs in the pulp and dual ECM scaffolds. The error bars represent mean ± SD (*n* = 4). No statistically significant change in cell attachment (day 1 time point) or rate of proliferation (slope of the lines) was observed for both DPSCs and HMSCs in the two types of scaffolds compared here.

Next, we evaluated the ability of the two types of ECM scaffolds to promote the attachment and proliferation of DPSCs and HMSCs. The purpose of this experiment was to evaluate if the presence of an endothelial ECM in conjunction with the pulp ECM would affect the proliferation of MSCs either positively or negatively. Results presented in **Figure 2** indicate that there was a linear increase in cell number of both DPSCs (**Figure 2A**) and HMSCs (**Figure 2B**) with increasing days in culture. In addition, results also indicated that the dual ECM scaffold did not negatively or positively influence the proliferation of both types of MSCs. No statistical significant change was observed in cell number or proliferation rate over time when the two scaffolds were compared.

### *In Vitro* Odontogenic Differentiation of MSCs

Our previous results show that pulp ECM scaffolds can promote the odontogenic differentiation of DPSCs and HMSCs *in vitro* by positively regulating the expression of genes that are involved in odontogenic differentiation (Ravindran et al., 2014a,b; Ravindran and George, 2015). Here, we evaluated if this ability was affected by the presence of an endothelial cell ECM in conjunction with the pulp ECM. Apart from confirming our initial findings, results presented in **Tables 1, 2** also indicated that the ability of the pulp ECM scaffolds to positively regulate the gene expression levels of pro-odontogenic growth factors, transcription factors and ECM proteins was not affected by the presence of the endothelial cell ECM. In addition, the expression levels of VEGF, a pro-angiogenic growth factor was increased in the dual ECM scaffold compared to the pulp ECM scaffold in both DPSCs and HMSCs.

**Table 1:** Odontogenic differentiation of DPSCs *in vitro.*

Genes	DPSC ECM	DPSC + HUVEC ECM
**Growth factors**		
BMP2	6.61 (0.006)	5.35 (0.004)
BMP9	19.19 (0.002)	12.31 (0.041)
GDF10	16.93 (0.0005)	11.28 (0.0025)
TGFB1	3.02 (0.004)	6.85 (4.36 E-7)
VEGFA	2.52 (1.5 E-3)	4.62 (0.0003)
**Transcription factors**		
RUNX2	2.09 (0.016)	2.73 (0.021)
OSX	3.80 (0.0009)	2.57 (0.035)
**ECM proteins**		
OCN	3.80 (0.017)	5.29 (0.012)
ALPL	2.67 (0.042)	No sig. change
DSPP	3.85 (0.005)	2.68 (0.027)


*Table representing the expression levels of odontogenic marker genes as fold change with respect to control scaffold after 2 weeks of DPSC culture within the pulp and dual ECM scaffolds. Statistical significance with respect to control is displayed as ox*P*-value from Student’s ox*t*-test in parentheses.*

**Table 2:** Odontogenic differentiation of HMSCs *in vitro.*

Genes	DPSC ECM	DPSC + HUVEC ECM
**Growth factors**		
BMP6	1.34 (0.016)	1.07 (0.041)
GDF10	6.41 (0.037)	7.16 (0.003)
TGFB1	3.92 (0.0004)	5.43 (0.0002)
VEGFA	2.73 (8.7 E-6)	5.65 (1.9 E-5)
**Transcription factors**		
RUNX2	2.38 (0.082)	2.69 (0.014)
OSX	Turned on	Turned on
**ECM proteins**		
OCN	3.34 (0.003)	2.81 (0.013)
ALPL	3.52 (0.0421)	3.72 (0.006)
DSPP	3.74 (0.001)	2.24 (0.014)
COL1	4.91 (0.001)	6.65 (1.9 E-5)


*Table representing the expression levels of odontogenic marker genes as fold change with respect to control scaffold after 2 weeks of DPSC culture within the pulp and dual ECM scaffolds. Statistical significance with respect to control is displayed as ox*P*-value from Student’s ox*t*-test in parentheses.*

### Influence of the Pulp and Dual ECM Scaffolds on DPSC Differentiation *in Vivo*

**FIGURE 3:**
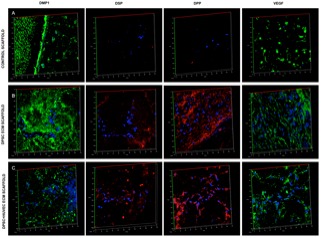
Fluorescence IHC of DPSC differentiation *in vivo*. Panels **(A–C)** are representative confocal micrographs of demineralized tooth root slice implants containing control **(A)**, pulp ECM **(B)**, and dual ECM **(C)** scaffolds embedded with DPSCs after 4 weeks of implantation subcutaneously in immunocompromised mice. The sections were immunostained for DMP1, DSP, DPP, and VEGF. Note the increased expression of DMP1, DSP, and DPP in the pulp and dual ECM scaffolds. Also note the increased presence of VEGF in the dual ECM scaffolds.

We evaluated the ability of the pulp and the dual ECM scaffolds to promote odontogenic differentiation of DPSCs as well as to promote vascularization *in vivo* in a tooth root slice subcutaneous implantation model. Results presented in **Figure 3** show that compared to control scaffolds (no ECM), DPSCs showed more robust odontogenic differentiation in the ECM scaffolds indicated by the expression levels of marker proteins DMP1, DSP, and DPP. Additionally, comparing the pulp and the dual ECM scaffolds, no discernible difference was observed in the expression of these proteins. The expression of the pro angiogenic growth factor VEGF was pronounced in both pulp and dual ECM scaffolds compared to the control and no discernible difference in expression was observed between the pulp and dual ECM scaffolds.

**FIGURE 4:**
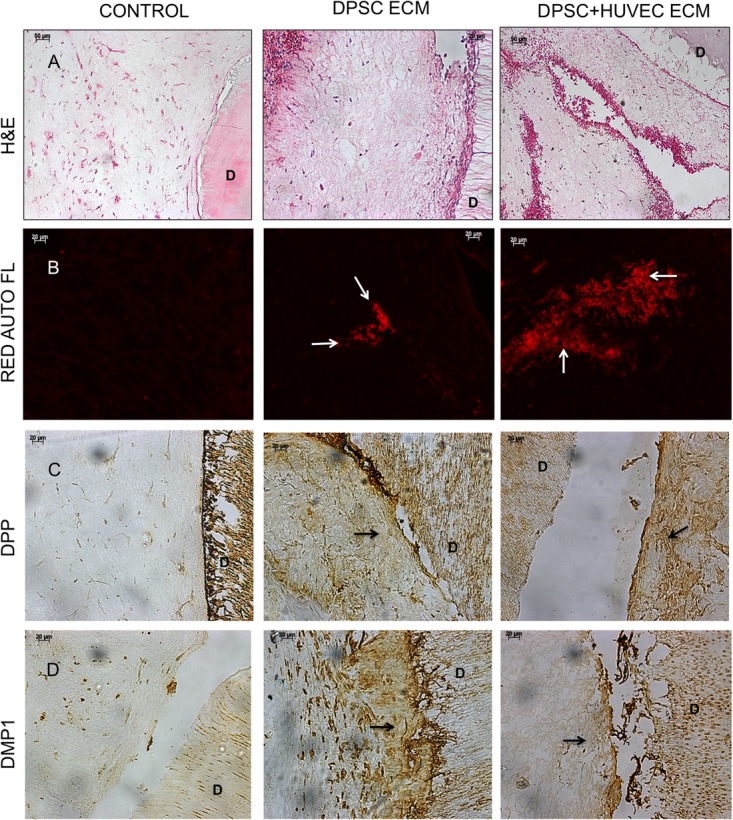
Histology and IHC of DPSC differentiation *in vivo*. Panels **(A–D)** are representative images of H&E **(A)**, red auto fluorescence **(B)**, DPP **(C)**, and DMP1 **(D)** stained sections from control, pulp ECM (DPSC ECM) and dual ECM (DPSC+HUVEC ECM) scaffolds. The white arrows in **(B)** indicate the presence of blood vessels containing RBCs. Note the robust vascularization in the dual ECM scaffolds. Black arrows in **(C,D)** point to positive staining of DPP and DMP1, respectively, in the pulp and dual ECM scaffolds at the pulp-dentin interface. In panels **(A–D)**, “D” represents dentin. The staining of DPP and DMP1 in the dentin serves as a positive control for the presence of these proteins and also as a control for exposure to the substrate.

Fluorescence IHC is suitable for observation of protein expression levels, as the scanning parameters of the confocal microscope can be maintained constant for obtaining comparative images. However, they are not ideal for observing spatial expression patterns of proteins. Therefore, in addition to the fluorescence IHC data presented in **Figure 3**, H&E staining as well as standard IHC staining was also performed. The results of these experiments are presented in **Figure 4**. H&E staining presented in **Figure 4A** shows representative images of general tissue morphology in all the groups. In addition, the dual ECM scaffolds promoted extensive vascularization of the scaffolds. The presence of blood vessels within the scaffolds was clearly observed (arrows in **Figures 4A,B**). **Figure 4B** shows representative fluorescence micrographs of red autofluorescence from the H&E stained sections. The red blood corpuscles (RBCs) fluoresce under standard RFP excitation and emission wavelengths. The images presented in **Figure 4B** indicate the presence of blood vessels (denoted by the presence of RBCs) in the pulp and dual ECM scaffolds. The Dual ECM scaffolds showed a more robust vascularization than the pulp ECM scaffolds. Quantitation was performed on six images spanning three random sections from different areas. Results indicated no significant vascularization of the control scaffolds. On the other hand, the dual ECM scaffolds showed 6.83 (*n* = 6, *P* < 0.01) fold increase in the area vascularized compared to pulp ECM scaffolds. **Figures 4C,D** are representative images of DPP and DMP1 IHC, respectively. Results show that compared to controls, both the pulp and dual ECM scaffolds induced more robust expression of both these proteins by the implanted DPSCs. Additionally, increased expression of both DPP and DMP1 at the pulp dentin interface was observed (marked by black arrows in both 4B and 4C). The expression levels of the proteins in natural dentin (represented by ‘D’ in the images) serve as both antibody and exposure control. Taken together, the results presented in **Figures 3, 4** indicate that both the pulp and the dual ECM scaffolds promote odontogenic differentiation of implanted DPSCs *in vivo.* However, the dual ECM scaffolds showed better potential for induction of vascularization compared to the pulp ECM scaffolds.

### Influence of the Pulp and Dual ECM Scaffolds on HMSCs Differentiation *in Vivo*

**FIGURE 5:**
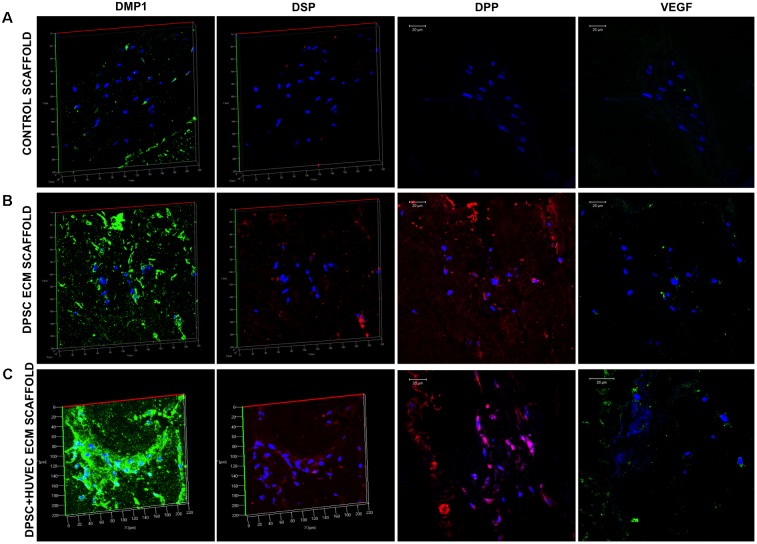
Fluorescence IHC of HMSC differentiation *in vivo.* Panels **(A–C)** are representative confocal micrographs of demineralized tooth root slice implants containing control **(A)**, pulp ECM **(B)**, and dual ECM **(C)** scaffolds embedded with HMSCs after 4 weeks of implantation subcutaneously in immunocompromised mice. The sections were immunostained for DMP1, DSP, DPP, and VEGF. Note the increased expression of DMP1, DSP, and DPP in the pulp and dual ECM scaffolds. Also note the increased presence of VEGF in the dual ECM scaffolds.

Although potent, DPSCs are MSCs that are available in limited quantities for autologous use. We have demonstrated previously that the pulp ECM scaffolds can induce odontogenic differentiation of HMSCs (Ravindran et al., 2014a). Here, we evaluated the ability of the pulp ECM scaffold alongside the dual ECM scaffolds to induce odontogenic differentiation of HMSCs *in vivo* and promote vascularization in the subcutaneous tooth root slice implantation model. **Figure 5** shows representative fluorescence IHC images for the expression levels of DMP1, DSP, DPP, and VEGF in control, pulp and dual ECM scaffolds. Results presented in **Figure 5** show that similar to the DPSCs, the pulp and dual ECM scaffolds promoted increased expression of DMP1, DSP, and DPP in HMSCs compared to no ECM control scaffolds. An increased expression of DMP1 was observed in the dual ECM group compared to both groups. On the other hand, a slightly decreased level of DSP and DPP expression was observed. Additionally, both experimental groups promoted only a modest increase in the expression levels of VEGF.

**FIGURE 6:**
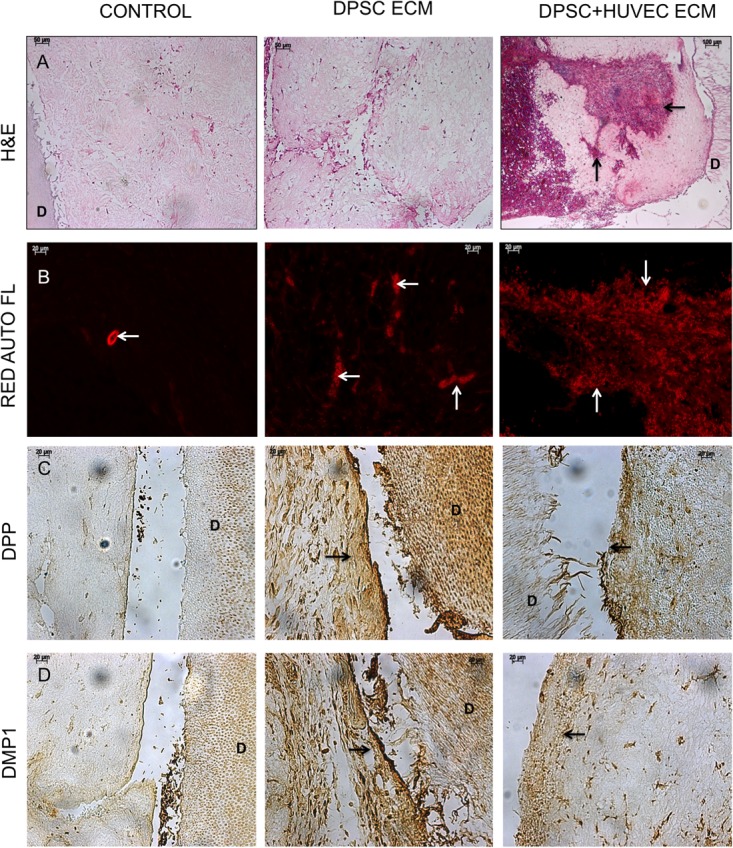
Histology and IHC of HMSC differentiation *in vivo*. Panels **(A–D)** are representative images of H&E **(A)**, red auto fluorescence **(B)**, DPP **(C)**, and DMP1 **(D)** stained sections from control, pulp ECM (DPSC ECM) and dual ECM (DPSC+HUVEC ECM) scaffolds. The black arrows in **(A)** and white arrows in **(B)** indicate the presence of blood vessels containing RBCs. Note the robust vascularization in the pulp as well as dual ECM scaffolds. Black arrows in **(C,D)** point to positive staining of DPP and DMP1, respectively, in the pulp and dual ECM scaffold at the pulp-dentin interface. In panels **(A–D)**, “D” represents dentin. The staining of DPP and DMP1 in the dentin serves as a positive control for the presence of these proteins and also as a control for exposure to the substrate.

Similar to the experiments involving DPSCs, H&E staining and standard IHC staining was performed on the samples as well. Results of these experiments are presented in **Figure 6**. H&E staining images presented in **Figure 6A** indicate that the overall tissue architecture. However, both the pulp and the dual ECM scaffolds were highly potent in combination in HMSCs to promote extensive vascularization of the pulp area. Fluorescence images presented in **Figure 4B** indicate that some vascularization was observed in the control scaffolds. However, both the pulp and the dual ECM scaffolds showed a much more robust vascularization pattern compared to the controls. Quantitation of vascularized area indicated that the dual ECM scaffolds showed 3.41-fold (*n* = 6, *p* < 0.01) increase. The IHC for DPP and DMP1 indicated that both the pulp and the dual ECM scaffolds triggered an increase in the expression levels of these marker proteins. Spatially, a more robust expression of these proteins was observed at the pulp-dentin interface in the pulp and dual ECM scaffolds and this phenomenon was absent in the control scaffolds. Taken together, the results presented collectively in **Figures 5, 6** indicate that the pulp and dual ECM scaffolds promote the odontogenic differentiation of HMSCs by promoting the expression of marker proteins DMP1, DSP, and DPP. The dual ECM scaffolds, in combination with HMSCs formed a potent force as inducers of vascularization.

## Discussion

Vascularization is an essential component of tissue regeneration. In the case of dental pulp, owing to the anatomy of the tooth, regenerative strategies need to emphasize proactive and efficient vascularization. Several recent studies have developed a wide range of methodologies to promote vascularization in the regenerating dental pulp tissue. Some of these approaches include the use of growth factors such as VEGF laden hydrogels to promote vascularization (Yadlapati et al., 2017). Although an elegant solution, this strategy poses a problem of dosage, delivery and ectopic activity. Therefore, there is an inherent hurdle to translational medicine.

Other approaches have evaluated pre-patterned hydrogels with embedded channels prefabricated into the hydrogel to facilitate vascularization when placed within the root canal space (Athirasala et al., 2017). However, these approaches too face several translational hurdles. The shape, size and architecture of the root canal space is not constant and varies considerably between the different teeth and also between individuals. Therefore, a high-resolution micro Ct image is required to cast a hydrogel of appropriate shape and size. In addition, once cast, it becomes a more difficult proposition to insert the hydrogel into the space without disrupting the embedded channels and also ensuring subsequent stability over time. These engineering challenges and the inability of this technique to enable mass production are debilitating hurdles for clinical translation.

In this study, we have focused on the development of a technique to generate hydrogels that can not only drive stem cell differentiation, but also promote robust vascularization. Here, we have developed a hybrid scaffold that contains the native pulp ECM in conjunction with the ECM of endothelial cells. The results presented in this study show that this dual ECM scaffold contains characteristic pulp ECM proteins as well as an increased amount of pro-vascular proteins in the form of VEGF, bFGF and von Willebrand factor. Our results show that the ability of the dual ECM scaffolds to promote the attachment and proliferation of MSCs is similar to that of the pulp ECM scaffolds indicating that the presence of the endothelial ECM does not hinder the ability of the scaffolds to promote MSC proliferation. In addition, this result also shows that the architecture of the scaffolds is not significantly altered to affect MSC attachment and proliferation rate.

Apart from the dental pulp stem cells, we have also focused on the ability of the scaffolds to induce odontogenic differentiation of bone marrow derived MSCs as well. Neural crest derived DPSCs are the best-suited cells for pulp regenerative applications (La Noce et al., 2014). However, their limited availability and reduced numbers pose hurdles toward their translatory potential. Therefore, other groups and us have ventured to evaluate the possibility of other MSC sources for pulp regenerative applications (Ishizaka et al., 2012). Published studies have also shown that stem cells isolated from the bone marrow, adipose tissue and placenta have similar epigenetic profiles (Aranda et al., 2009) making choice amongst them simpler.

Our results indicate that the dual ECM scaffolds were able to initiate the odontogenic differentiation of both DPSCs and HMSCs *in vitro*. This was evident from the gene expression data that indicated a positive regulation of pro-odontogenic genes with statistical significance. In addition to this, a comparison between the pulp ECM scaffold and the dual ECM scaffold performance indicated that the dual ECM scaffolds also triggered an increased expression of pro-angiogenic genes such as VEGF more than the pulp ECM scaffolds. When implanted subcutaneously in a simulated root slice model, the dual ECM scaffolds triggered the odontogenic differentiation of both DPSCs and HMSCs. Confocal and light microscopy showed an increased expression of marker proteins DSP, DPP, and DMP1 in both pulp and dual ECM scaffolds indicating the initiation of odontogenic differentiation. The results from this model also indicated that apart from initiating cellular differentiation, the pulp ECM scaffolds also promoted the secretion of DMP1 and DPP at the interface of the pulp and dentin. This is clearly observed in the histology and IHC data. This interesting behavior indicates the ability of the pulp and the dual ECM scaffolds to trigger and promote the formation reparative dentin at the pulp-dentin interface.

All of these results taken together indicate that the dual ECM scaffolds performed on par with the pulp ECM scaffolds *in vitro* and *in vivo* in terms of MSC differentiation and reparative dentin formation. However, our results indicated that the dual ECM scaffolds were far superior in their ability to promote vascularization. Qualitative and quantitative histology data indicated the presence of robust blood vessels within the dual ECM scaffolds with the presence of RBCs indicating active blood flow. Particularly, the combination of the dual ECM scaffold with HMSCs proved to be the most potent in terms of vascularization. Overall, the results indicated that bone marrow MSCs promoted more active vascularization in both control and experimental scaffolds compared to DPSCs in our model. Further studies on the mechanistic aspects of ECM triggered pathways are required to understand and evaluate this result.

Overall, the results from this study standardized a methodology to generate hybrid pulp/endothelial ECM containing biomimetic scaffolds. This dual ECM scaffolds promoted MSC attachment, proliferation and odontogenic differentiation *in vitro* and *in vivo*. From a translation perspective, we envision that these dual ECM scaffolds can be mass-produced in the form of sheets using bioreactors and stored at room temperature in lyophilized form similar to clinical grade collagen sponges. These can be cut and hydrated to fill the root canal spaces *in situ* in clinics without the need for pre-fabrication or complex growth factor loading and release profiles. As the biomimetic scaffolds contain cell-secreted matrix, they are composed of tissue-specific human proteins in physiologically relevant quantities and hence the possibility of adverse immunological complications and rejection are greatly reduced paving the way for successful clinical translation. Further experiments in orthotropic sites in larger pre-clinical animal models are required to validate these properties.

## Author Contributions

C-CH performed and planned several *in vitro* experiments and performed the animal surgeries and histological staining. RN generated the scaffolds, maintained the cells used, and performed most of the qPCR analyses. NW maintained primary cells and performed qPCR analysis of HMSCs on the pulp and dual ECM scaffolds. SR conceptualized the study, planned the series of experiments, organized the data, and wrote the article.

## Conflict of Interest Statement

The authors declare that the research was conducted in the absence of any commercial or financial relationships that could be construed as a potential conflict of interest. The reviewer VT and handling Editor declared their shared affiliation.
